# Growth hormone-releasing hormone, growth hormone, and neuroendocrine tumors^☆^

**DOI:** 10.1016/j.jcte.2026.100458

**Published:** 2026-09-11

**Authors:** Maham Shahid, Christian A. Koch

**Affiliations:** Division of Endocrinology, Diabetes & Metabolism The University of Florida, Jacksonville, United States

**Keywords:** Growth hormone, Growth hormone releasing hormone, Neuroendocrine tumors, Ectopic acromegaly, Ectopic hormone syndrome, Somatostatin receptor, Somatostatin analogue

## Abstract

Neuroendocrine tumors (NETs) are known to produce growth hormone (GH) and growth hormone-releasing hormone (GHRH) leading to acromegaly. Ectopic production of these hormones can lead to ectopic acromegaly which is very rare. Ectopic acromegaly secondary to GHRH has been studied more extensively than GH-related ectopic acromegaly. Given the extreme rarity of these neoplasms, comprehensive guidelines for diagnosis and management of these NETs are currently lacking. This review summarizes the literature on clinical presentation, challenges in diagnosis and management of these NETs. The diagnosis of ectopic acromegaly is often difficult as clinical features do not differ from eutopic acromegaly, and we discuss herein when the clinical suspicion should be held high. GHRH serum concentrations are a useful diagnostic tool when ectopic production is suspected. Once the ectopic source is identified using imaging modalities including advanced molecular imaging, the choice of treatment is surgical resection of the respective NET. Other therapies include use of somatostatin receptor analogues and chemo- or immuno-therapies as an adjunct or in refractory cases.

## Introduction

Neuroendocrine tumors (NET) are a heterogeneous group of tumors that can arise from various tissues. Over the years, increasing awareness of these neoplasms and advanced imaging technologies have led to an uprising detection rate [1], [2], [3], [4]. The Canadian, European, and North American Neuroendocrine Tumor societies provide updated guidelines on diagnosing and treating these tumors [3], [4], [5], [6]. NETs can secrete a variety of peptides and hormones, including GH and GHRH [7], [8], [9], [10], [11], [12], [13], [14], [15], [16], [17], [18], [19], [20], [21], [22], [23]. Somatostatin receptor-based imaging is typically used in diagnosing and staging of NETs. In that regard, it is important to be aware of somatostatin receptor expression and tracer uptake in certain tissues in the absence of a NET [24]. Treatment with radiolabeled somatostatin analogs has evolved in the management of patients with inoperable or metastasized NETs and Lutetium 177-DOTATATE (^177^Lu-DOTATATE) peptide receptor radionuclide therapy appears to be effective in gastroenteropancreatic metastatic NETs [25]. The use of immunotherapy has increased for various cancers and NETs with various ongoing trials [26]. The incidence and discovery of pulmonary NETs is increasing with somatostatin analog treatment remaining the backbone of therapy [27].

### Illustrative case

**Fig. 1:**
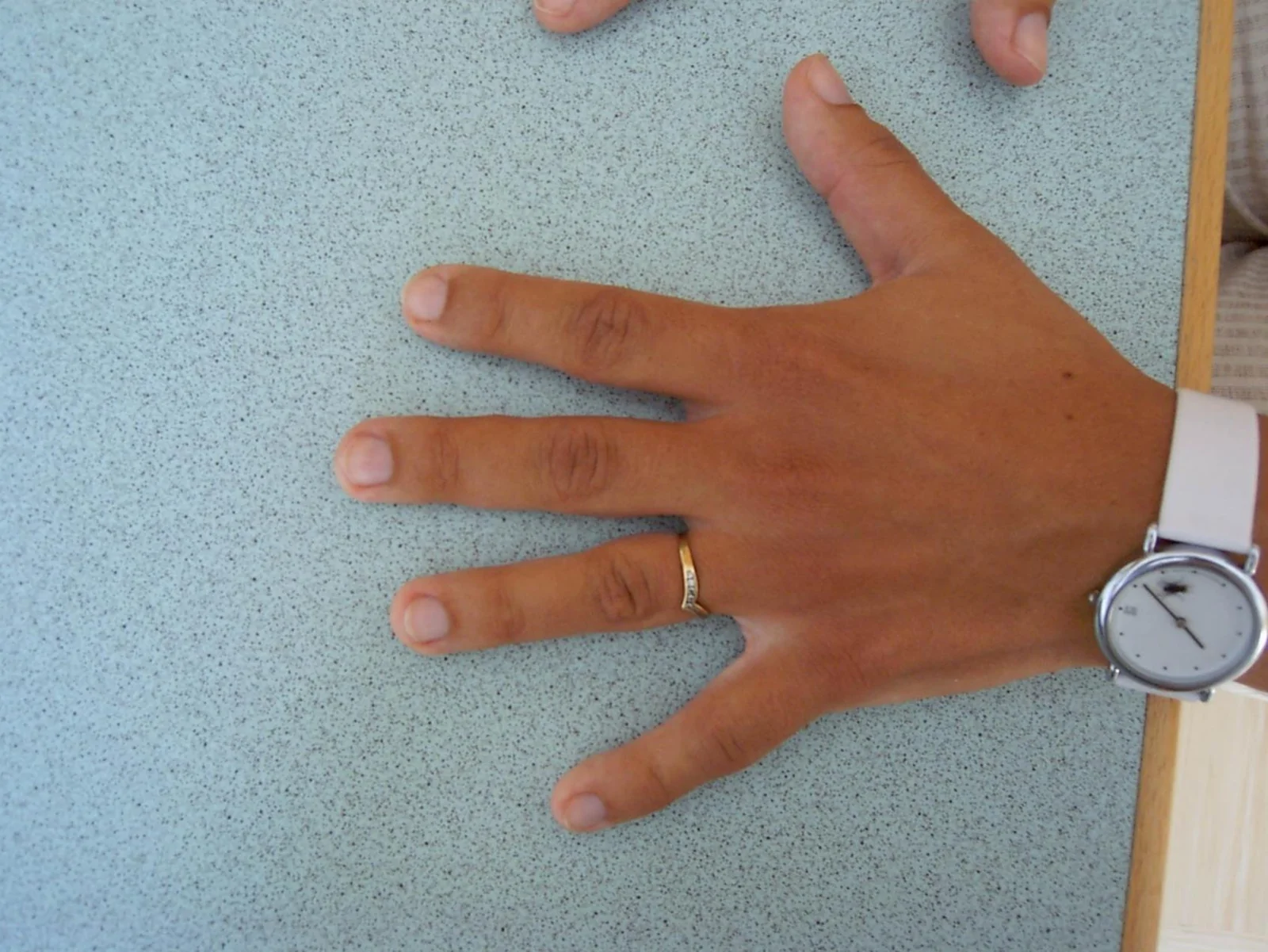

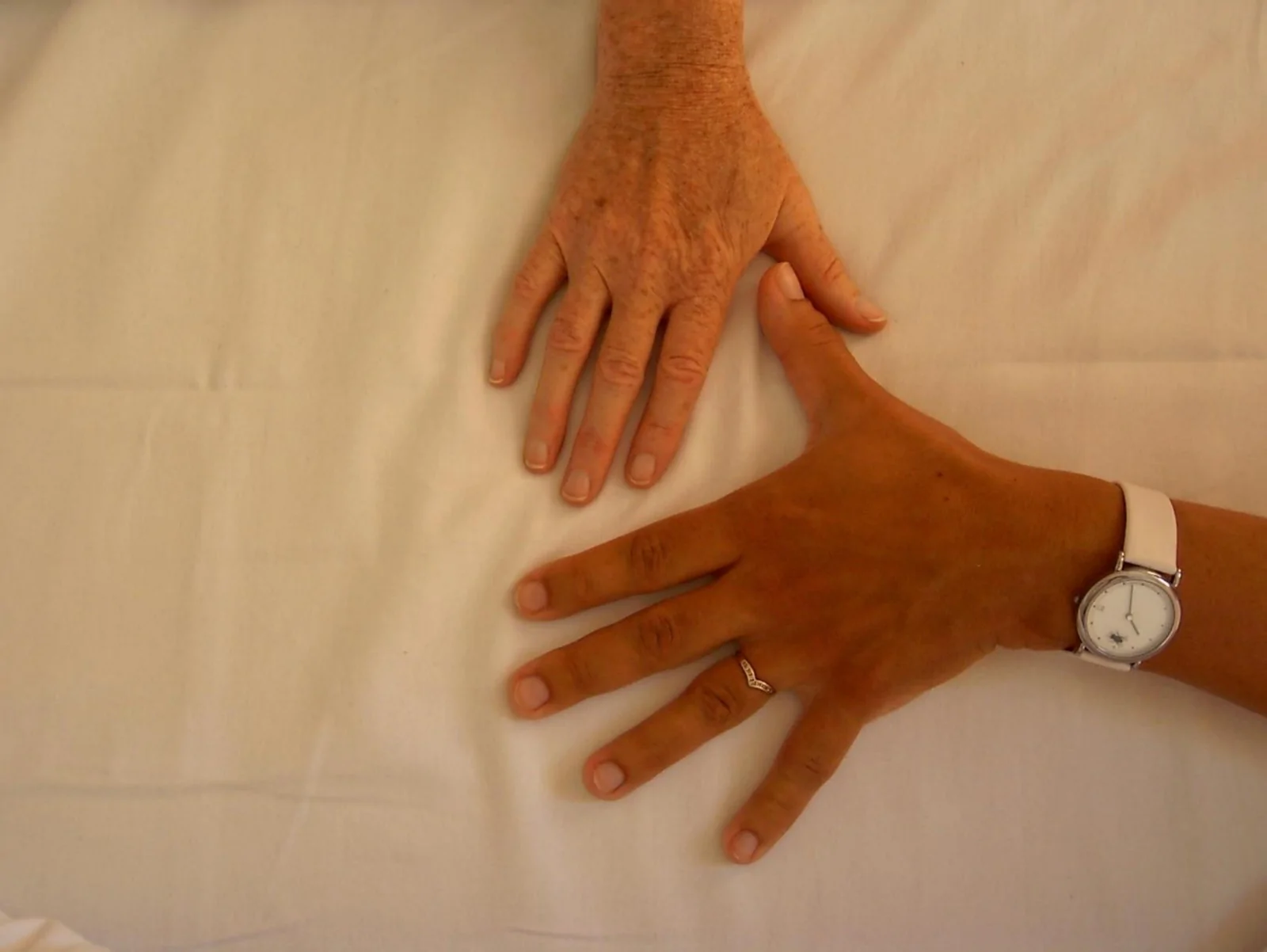

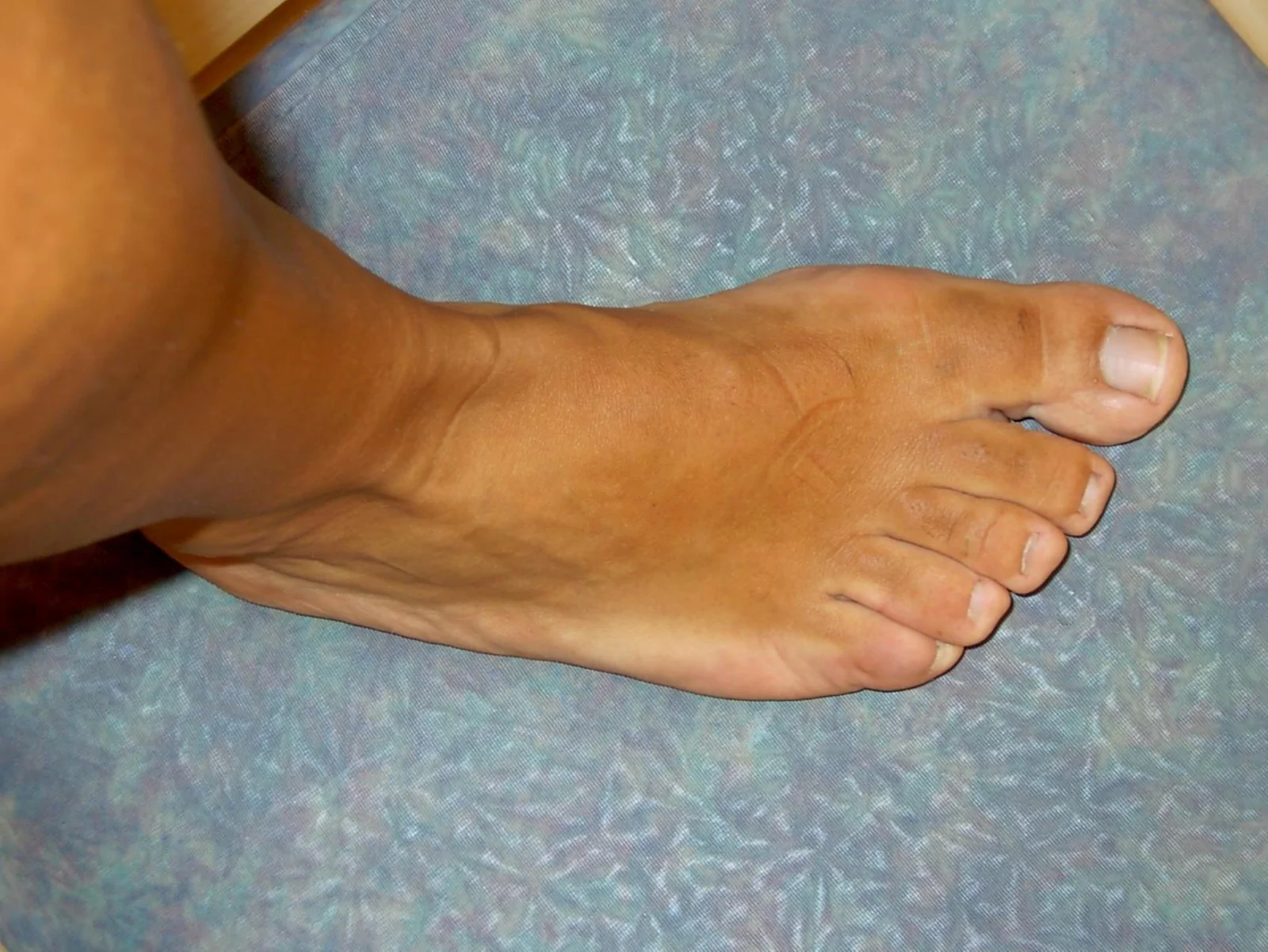
1a. Left hand of female watchmaker showing diffuse enlargement and broadening, increased soft-tissue bulk, and widened inter-digital spaces, consistent with acromegalic features. 1b. Left hand of the female watchmaker with acromegaly wearing a white watch and hand of another woman without acromegaly. 1c. Right foot showing diffuse enlargement with broadening of the forefoot, increased soft-tissue bulk, and thickened toes consistent with acromegalic changes.

A 40-year-old female watchmaker presented for evaluation of longstanding multinodular goiter [13]. She reported having soft tissue swelling, profuse sweating, oligomenorrhea, excessive hair growth, and noticed an increase in U.S. shoe size from 8 to 10.5. She had spade-like fingers and broad feet (Fig. 1a, b, c).

**Fig. 2:**
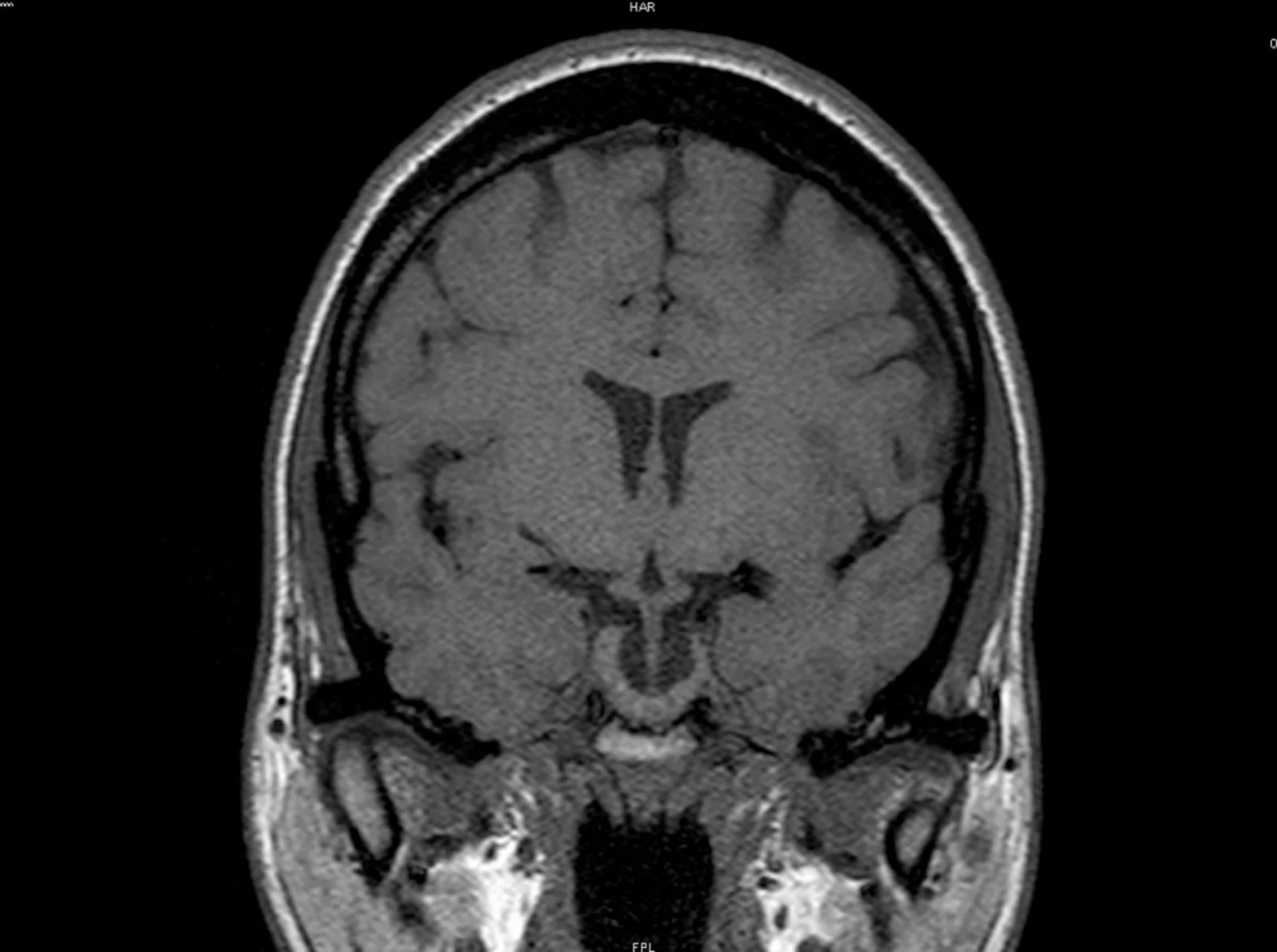
Pituitary MRI with no pituitary lesions identified.

She denied headaches, visual disturbance, chest pain, polyuria, and fatigue. On physical examination she appeared acromegalic with a large tongue, prognathism, and coarse facial features. She had a height of 173 cm and a weight of 95 kg. She had a goiter with a volume of 100 ml (normal, <20 ml) and a blood pressure of 145/80 mmHg. Laboratory testing revealed normal thyroid function, normal liver enzymes, and normal renal function. Prolactin was elevated at 41 μg/L (Reference range 4–20 μg/L). Insulin-like growth factor-1 (IGF-1) measured in serum was 648 μg/L (standard deviation score 3.41 and elevated for age and sex; normal: −2 to +2). A 75-g oral glucose tolerance test (75 g OGTT) showed no suppression of GH with a minimal GH of 24 μg/L (Reference range 0.01–8.0 μg/L). To search for a GH-secreting pituitary adenoma, magnetic resonance imaging (MRI) was performed and did not demonstrate a pituitary adenoma (Fig. 2).

**Fig. 3:**
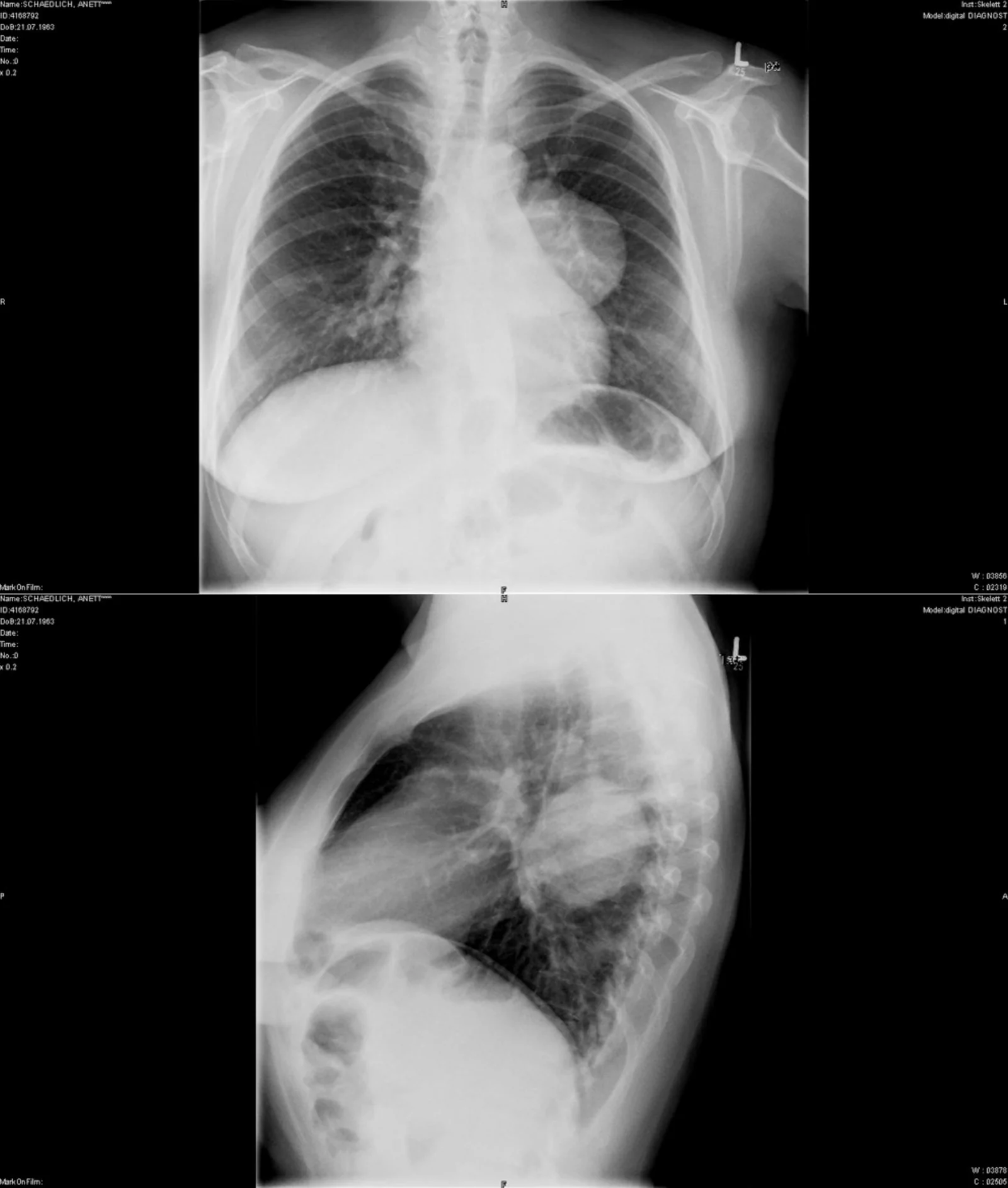
Chest x-ray showing left thoracic mass.

Routine chest x-ray (typically performed on admission to the hospital) showed a left thoracic mass measuring 7 × 5 cm (Fig. 3a, b).

**Fig. 4:**
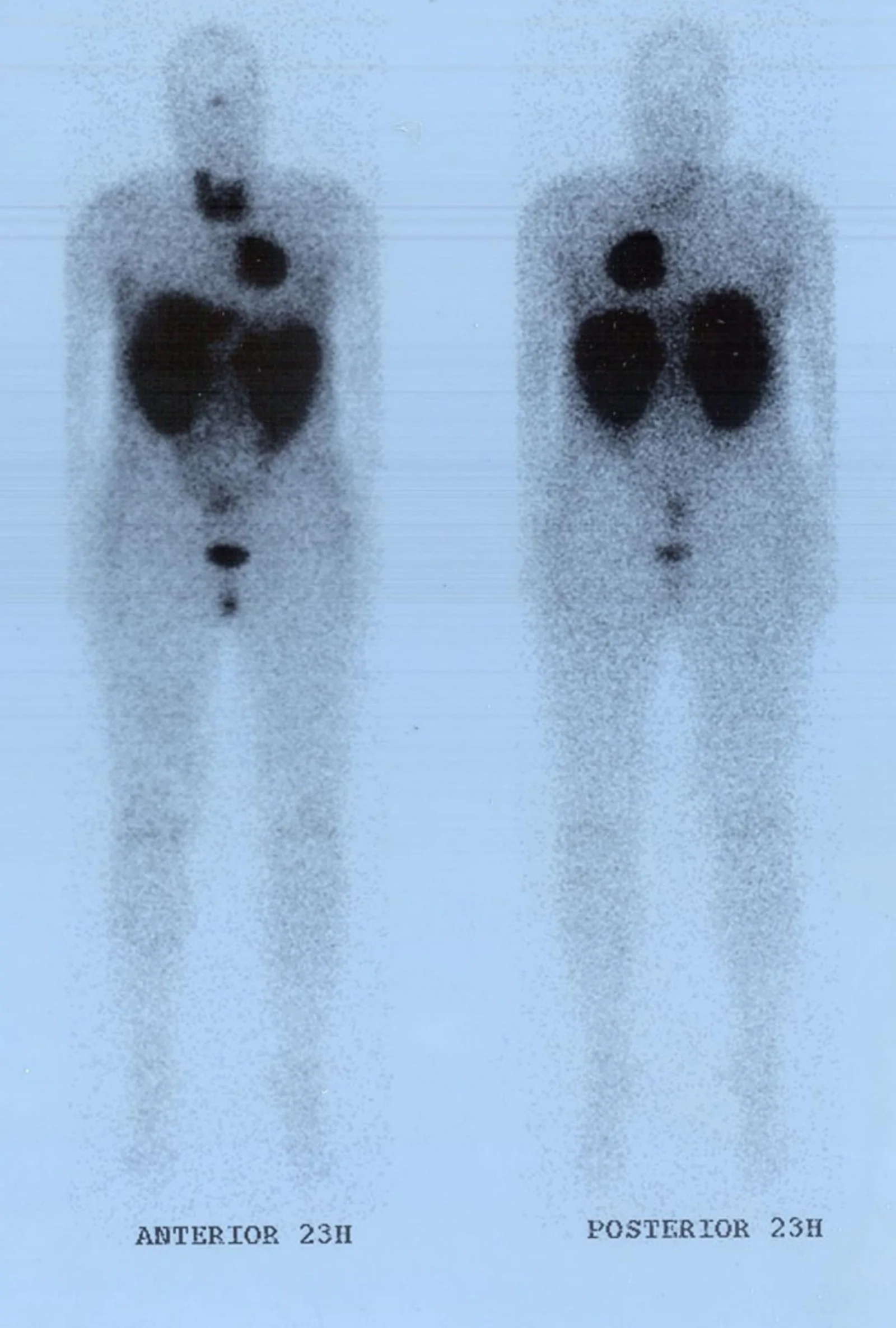
111Indium-pentetreotide scintigraphy showing increased tracer uptake projecting to the left lung (pathologic) and thyroid region (physiologic).

To further evaluate the reason for acromegaly and GH excess, somatostatin receptor scintigraphy was performed showing tracer uptake projecting to the left lung (pathologic) and physiologic uptake in the thyroid region due to the presence of somatostatin receptors in thyroid tissue (Fig. 4).

**Fig. 5:**
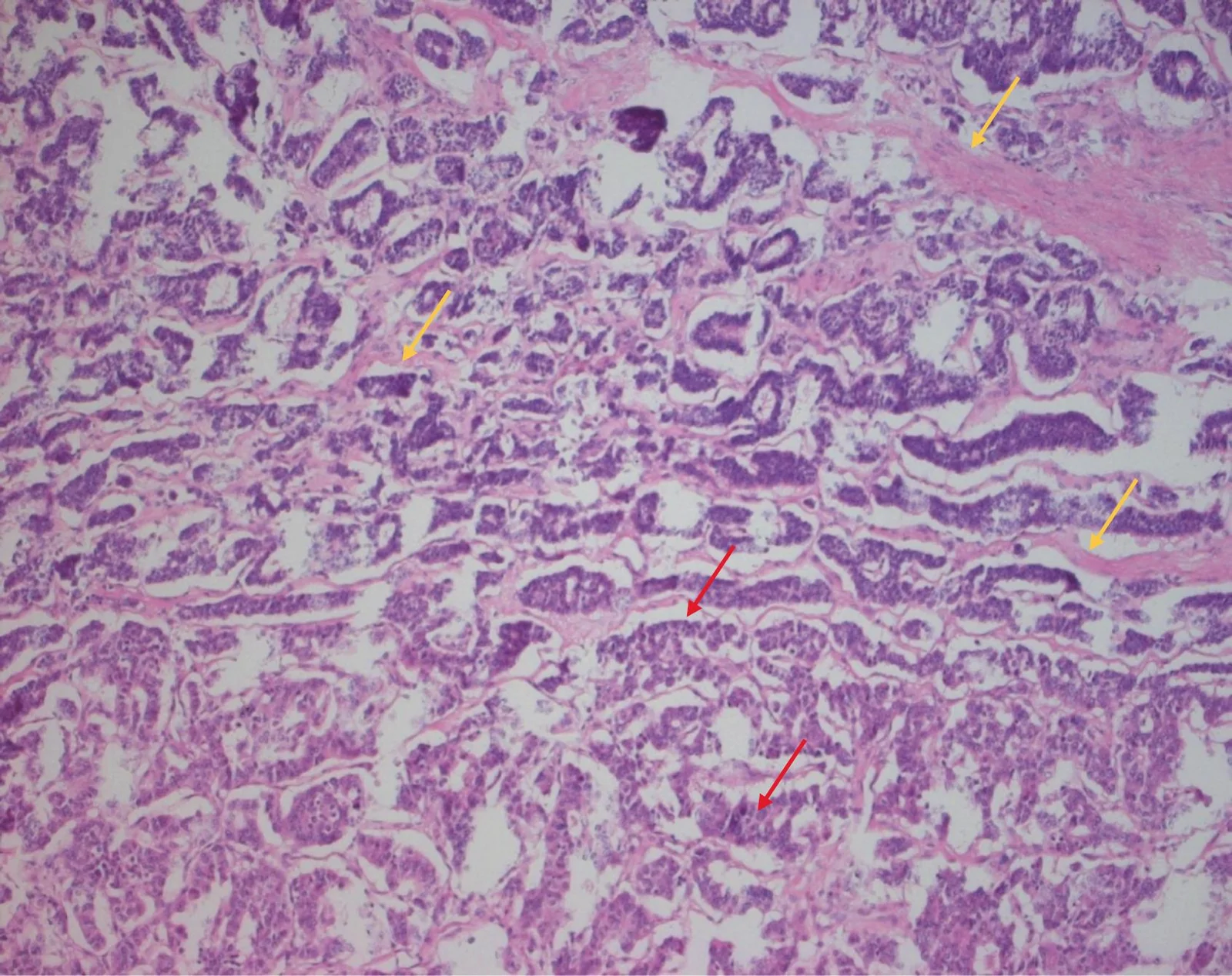

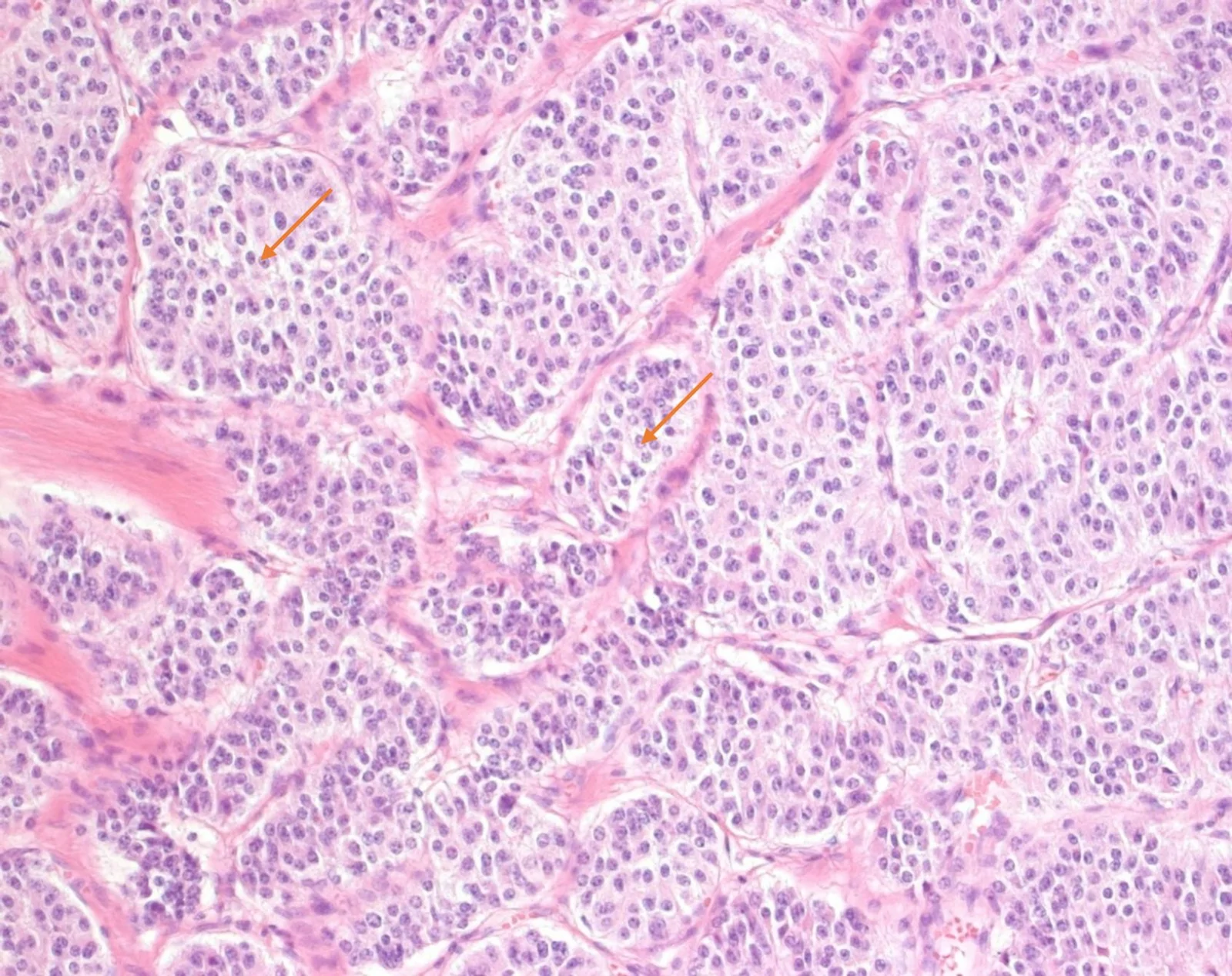
5a. Typical carcinoid NET of the lung with trabecular growth pattern (red arrows) separated by fibrous septae (yellow arrows). 5b. Tumor cells containing nuclei with visible nucleoli and foamy cytoplasm (orange arrows).

The patient underwent complete lung tumor resection. Histopathology revealed a typical NET with a Ki-67 index <2%. (Fig. 5a,b). Genetic analysis was not performed.

Postoperatively, the woman reported marked improvement of soft tissue swelling and of sweating. She also noticed her goiter to be smaller. Menstrual cycles normalized and excessive hair growth declined. She no longer needed antihypertensive drug therapy. Follow-up somatostatin receptor scintigraphy and chest x-rays did not show any evidence of tumor recurrence. The results of the bronchoscopy were normal. Prolactin was measured within normal range. IGF-1 serum concentrations (age and gender specific reference ranges) more than three months after tumor resection were normal. 75 g OGTT showed a GH of 0.99 μg/L six weeks and three months after tumor resection. Plasma chromogranin A and neuron specific enolase were negative.

We were not able to measure GHRH in plasma/serum but immunohistochemical staining of the lung tumor specimen revealed cytoplasmic expression of GHRH, making ectopic GH secretion as a cause of acromegaly less likely.

## Epidemiology

Acromegaly is considered a rare disease, with a yearly incidence of less than 0.2–1.1 cases per 100,000 people, and the prevalence is about 60 cases per 1 million [21], [28]. 99% of cases are eutopic consistent with a pituitary adenoma, and extra-pituitary or ectopic sources account for less than 1% of all cases [21]. The most common cause of ectopic acromegaly is peripheral GHRH-secreting tumors while the rarest is peripheral GH-secreting tumors [22], [23].

The age distribution ranges from 14 to 77 years and the mean age at the time of diagnosis is comparable in men and women (43.9 vs 41.7 years) with a diagnostic delay seen in men by approximately 3.5 years as compared to women (9.9 vs 6.3 years) [22]. This disparity from the time of onset of symptoms until diagnosis in men and women is consistent with the characteristics of pituitary acromegaly [29]. Acromegaly caused by a GH-secreting pituitary adenoma usually shows a balanced female-to-male distribution, however, for ectopic acromegaly due to GHRH secretion up to 70% of the cases were reported as female patients [22].

### Historical aspects of ectopic acromegaly

Ectopic acromegaly has been reported in the literature as early as the 1950s. To the best of our knowledge, in the late 1950s Altmann and Shutz reported the first case of acromegaly refractory to pituitary irradiation in a 47-year-old female with bronchial carcinoid whose acromegalic symptoms improved after resection of the bronchial mass [30]. Only a few years later in 1961, Weiss and Ingram reported a similar case of ectopic acromegaly in a 45-year-old male patient with bronchial carcinoid [31]. Human GH activity was described in pharyngeal hypophyses from embalmed cadavers in 1968 [32]. Subsequent reports showed clinical GH excess in patients with tumors of sphenoid or parapharyngeal sinuses [33], [34]. Frohman et al. isolated a peptide with GH-releasing activity from lung and pancreatic NETs in 1980 [35]. Soon thereafter, cases of ectopic acromegaly associated with pancreatic NETs were reported [36], [37].

### Types of ectopic acromegaly with respect to origin and hormonal secretion

Ectopic acromegaly is secondary to either extra-pituitary secretion of GH or GHRH from NETs. These can further be categorized into tumors that are located intra-cranially and those which are located peripherally [23]. Therefore, tumors causing ectopic acromegaly can be classified into following four types:

1) Central ectopic GH-secreting tumors.
2) Peripheral GH-secreting tumors.
3) Central ectopic GHRH-secreting tumors.
4) Peripheral GHRH-secreting tumors.

### Ectopic GH-secreting Tumors

21 cases of ectopic acromegaly due to GH secreting tumors have been described in the literature until 2022 [22], [23]. We performed a PubMed and Google Scholar search using the terms ‘ectopic’, ‘acromegaly’, ‘growth hormone’, ‘GH’, and found a total of 30 cases of acromegaly caused by an ectopic source of GH until 2025 including those reported by previous authors. This includes 23 central GH-secreting extra-pituitary tumors and 7 cases of peripheral GH-secreting tumors. GH-secreting ectopic tumors were found to have female predominance with 16 cases reported in women and 12 in men, and gender is unknown in two of the reports.

Out of the 23 intracranial ectopic tumors causing acromegaly, 12 were found in the sphenoid sinus including 1 in the bony inter-sphenoid septum and 6 tumors presented as clival masses [33], [34], [38], [39], [40], [41], [42], [43], [44], [45], [46], [47], [48], [49], [50], [51], [52], [53]. Tajudeen et al. reported a series of 9 patients in 2016 with ectopic pituitary adenomas arising from sphenoid or clival regions. Only one of these cases presenting with a clival lesion had ectopic acromegaly due to excess GH secretion [51]. Endo T et al. reported a case of ectopic GH-secreting isolated clival tumor separated from the normal pituitary gland that underwent malignant transformation in a couple of years [53]. One of the reported cases had an ectopic source located in both sphenoid sinus and clivus [54]. Two cases of ectopic suprasellar GH-secreting adenomas have been reported [55], [56]. Zhu et al. in 2020 reported 13 cases of suprasellar pituitary adenomas; two of these were GH-secreting with the clinical syndrome of acromegaly and only one of these two patients had an isolated suprasellar ectopic GH-secreting macroadenoma while the other patient had a large primarily pituitary adenoma with extension into the suprasellar cistern [56]. Only 1 case of an ectopic GH-secreting tumor of cavernous sinus separate from normal pituitary has been reported thus far [57]. A case of ectopic GH secretion from a temporoparietal meningioma with normal pituitary has also been reported [58]. Most of these intracranial ectopic tumors had normal pituitary on imaging with 8 cases demonstrating Empty Sella (ES) and only 1 case reported by Appel et al. revealed presence of a non-functioning pituitary microadenoma [49]. In patients with clinically and biochemically proven acromegaly, no initial clinical suspicion of ectopic source, and imaging demonstrating pituitary adenoma, transsphenoidal pituitary adenoma resection may be performed. It is when post-operative histopathology and immunohistochemistry reveal a non-functioning nature of the pituitary adenoma with no residual adenoma on imaging, an ectopic source of acromegaly should be investigated.

Among the extracranial sources of GH-related acromegaly, 4 cases with ectopic GH production in lungs, 1 in pancreas, 1 in ovary, and 1 case of non-Hodgkin lymphoma (NHL) have been reported [37], [59], [60], [61], [62], [63], [64].

Table 1 shows ectopic GH-secreting tumors reported until December 2025 with respect to origin of the tumors, gender distribution and findings of pituitary imaging.

**Table 1:** Origin, gender distribution, and pituitary imaging of Ectopic GH-secreting tumors until 2025. F: Female; M: Male; ES: Empty Sella; N/A: Not available.

Ectopic GH-secreting tumors									
Tumor site (*n* = 30)			Number of tumors (n)	Gender			Pituitary imaging		
				F (n)	M (n)	N/A	Normal (n)	ES (n)	Other
Intracranial (*n* = 23)	Sphenoid sinus		12	7	4	1	6	6	–
	Clivus		6	3	3	–	3	2	Microadenoma (non-functioning)
	Sphenoid Sinus and Clivus		1	1	0	–	1	–	–
	Ectopic suprasellar		2	1	1	–	2	–	–
	Cavernous Sinus		1	1	0	–	1	–	–
	Temporoparietal Meningioma		1	-	-	1	1	–	–
Extracranial (*n* = 7)	Lung	Bronchial Carcinoid	3	1	2	–	3	–	–
		Adeno-carcinoma	1	-	1	–	–	–	N/A
	Pancreas		1	-	1	–	1	–	–
	Non-Hodgkin Lymphoma		1	1	-	–	1	–	–
	Ovarian mature cystic teratoma		1	1	-	–	1	–	–

### Ectopic GHRH-secreting Tumors

Since the discovery of GHRH in the early 1980s, more than 150 cases of ectopic acromegaly due to central and peripheral secretion of GHRH have been reported among which the most frequent locations are the lung (bronchial) and pancreatic NETs [22], [36], [65], [66]. This contrasts with ectopic GH-secreting tumors where intracranial ectopic production is at least 3 times more common than peripheral sources as described in Table 1.

Among the 127 cases of GHRH-related ectopic acromegaly reported from 1982 to 2021 by Zendran et al., central GHRH-secreting tumors comprised of 27 cases with all tumors located in the sellar region, most of which were found to be mixed gangliocytoma-pituitary adenomas on histopathology while less common causes included isolated gangliocytomas and pituitary adenomas, and lymphoma with somatotroph hyperplasia [22]. As for 78.7% of the cases due to peripheral GHRH-secretion, the majority originated in the lungs (50%) and pancreas (35%) [22]. Over half of the cases of the pancreatic NETs were associated with Multiple Endocrine Neoplasia type 1 (MEN1) syndrome and were more likely to co-secrete other hormones such as insulin, gastrin, calcitonin, somatostatin, glucagon and pancreatic polypeptide, while in a few cases multiple pancreatic tumors were also identified [22], [67], [68], [69], [70], [71]. Other rare locations included gastro-intestinal tract (5%), adrenal gland (3%), thymus (2%), liver (1%), mediastinum (1%) and retroperitoneum (1%), in addition to 2% of the cases where the source remained unidentified [22], [72], [73], [74], [75], [76].

We performed a PubMed and Google Scholar search using the terms ‘ectopic’, ‘acromegaly’, ‘growth hormone releasing hormone’, ‘GHRH’, from 2022 to 2025 and 6 more cases of ectopic GHRH-secreting tumors were found, including a series of 3 cases with ectopic GHRH-secreting bronchial NETs in Russia, 2 separate cases of bronchial NETs with one associated with pituitary apoplexy, and a case of GHRH-secreting pancreatic NET in a MEN1 patient with pituitary hyperplasia. [77], [78], [79], [80]. During our literature search no new reports of central GHRH-secreting ectopic tumors during this period were identified.  Table 2 shows a compilation of 133 cases of ectopic GHRH-secreting tumors including 127 cases reported by Zendran et al. from 1982 to 2021 and the 6 additional cases reported from 2022 to 2025 as presented in this review.

**Table 2:** Origin of Ectopic GHRH-secreting tumors between 1982 and 2025, table modified from Zendran et al. 2022. GIT = gastrointestinal tract.

Ectopic GHRH-secreting Tumors			
Tumor site (*n* = 133)		Tumor Type	n
Intracranial (*n* = 27)	Sellar region	Mixed gangliocytoma-pituitary adenoma	21
		Gangliocytoma	3
		Pituitary adenoma	1
		Lymphoma with somatotroph hyperplasia	1
		Unidentified	1
Extracranial (*n* = 106)	Lung	Typical & atypical bronchial carcinoid	52
		Other	2
		Lack of histopathological examination	1
	Pancreas		36
	GIT		5
	Adrenal		3
	Thymus		2
	Liver		1
	Mediastinum		1
	Retroperitoneum		1
	Unidentified		2

Other tumors that are shown to express GHRH in vitro as assessed by immunohistochemistry (IHC) and radioimmunoassay (RIA) using anti-GHRH antibodies include parathyroid and medullary thyroid carcinomas, small cell lung carcinomas, breast and endometrial carcinomas, hypothalamic hamartomas, choristomas and ganglioneuromas, however, the presence of clinical acromegaly in these cases was not seen or is unknown [22], [66], [81]. This is thought to be due to insufficient release of GHRH from these tumors to stimulate the pituitary gland to produce excess GH or due to assay cross-reactivity of GHRH without biologic activity [66].

### Clinical features of ectopic acromegaly

Ectopic acromegaly presents similar to eutopic acromegaly with typical clinical symptoms of GH excess ranging from acral enlargement, soft tissue swelling, sweating, thickening of the skin, headache, arthralgia, carpal tunnel syndrome, and hyperhidrosis to advanced metabolic complications such as hypertension, type 2 diabetes mellitus, and sleep apnea syndrome [22], [81].

### When to suspect an ectopic source of acromegaly

Ectopic acromegaly should be suspected in the following circumstances:

• In individuals with biochemically confirmed acromegaly and normal pituitary gland on imaging.
• In patients with biochemically proven acromegaly who do not improve after successful pituitary surgery [22]. In such cases, a re-evaluation of the pituitary tissue, IHC, serum GHRH measurement, or somatostatin receptor scintigraphy may be obtained [81].
• In patients with a known non-pituitary tumor presenting with acromegaly, ectopic production of GH or GHRH should be suspected [22].

### Biochemical diagnosis of ectopic acromegaly

The initial biochemical profile of ectopic acromegaly is identical to the one of pituitary acromegaly. These tests include determination of IGF-1 levels, GH levels, and/or demonstration of non-suppression of GH levels after a 75 g OGTT.

The 2014 Endocrine Society guidelines recommend using IGF-1 levels normalized for age but not for sex, and lack of suppression of GH to <1 μg/L during an 75 g OGTT if necessary [82]. According to the 14th Acromegaly Consensus Conference held in Italy in 2022, IGF-1 levels greater than 1.3 times the upper limit of normal for age in the presence of characteristic clinical signs of the disease are diagnostic [83]. For equivocal results the consensus panel recommends IGF-1 serum levels to be repeated or additionally utilizing a 75 g OGTT [83]. It is to be noted that GH after a 75 g OGTT may also show a paradoxical increase [21].

In individuals with biochemically confirmed acromegaly and normal pituitary gland on imaging, the next step should be to evaluate for ectopic acromegaly by obtaining circulating GHRH plasma/serum levels which are usually undetectable in pituitary acromegaly. GHRH laboratory tests are commercially not widely available but can be done via Mayo Clinic Laboratories at Inter Science Institute (Test Name: Growth Hormone-Releasing Hormone, GHRH, also called GH-RH, GHRF, or GRF, Test ID: FGHRH, Methodology: Enzyme Immunoassay (EIA), Reference Range: 5–18 pg/mL, Turnaround time: approximately 12–14 days, Current Procedural Terminology (CPT) Code: 83520, weblink: https://www.mayocliniclabs.com/test-catalog/overview/75910) and https://www.interscienceinstitute.com/assay/growth-hormone-test/. It is also available as an esoteric test via Quest Diagnostics (Test Name: Growth Hormone Releasing Hormone, GH RH or GRF, Quest Test Code: 39336, Methodology: EIA, CPT Code: 83520, website link: https://testdirectory.questdiagnostics.com/test/test-detail/39336/growth-hormone-releasing-hormone-gh-rh-or-grf?cc=MASTER). GHRH levels vary widely between laboratory tests/assays and range from hundreds to thousands-fold normal values. Generally, a GHRH concentration > 250–300 ng/L is considered very suggestive of ectopic acromegaly [66], [79], [81], [84]. Due to variability in the degree of differentiation of NET, the GHRH levels do not correlate and should not be used to determine the extent of disease burden or extension of the tumors [85].

Secretion of GHRH by NETs can also be confirmed by histopathological examination by positive immunostaining or RIA with anti-GHRH 1–40 or anti-GHRH 1–44 antibodies, however, this testing is not widely available [86], [87]. Other less frequently used diagnostic methods include high performance liquid chromatography, ion exchange chromatography, detection of messenger RNA for GHRH in the tumor by polymerase chain reaction, and measurement of the arterio-venous gradient of GHRH across the tumor [88], [89], [90]).

Due to marked difference in the incidence ectopic GH-secreting tumors should be suspected only when ectopic GHRH-secretion is ruled out.

Co-existence of Hyperprolactinemia and Other Hormones & Effect on Pituitary Morphology.

Interestingly, the co-existence of hyperprolactinemia is not uncommon and occurred in our illustrative case [13]. Zendran et al. reported that 30% and 57% of cases of ectopic GHRH-related acromegaly with a primary tumor in the lung and pancreas, respectively, had simultaneous hyperprolactinemia [22]. The etiology of co-existing hyperprolactinemia is unclear but is thought to be the result of expected trophic effects of GHRH on the somatotrophs causing pituitary hyperplasia which may lead to compression of the pituitary stalk [66], [81]. This pituitary hyperplasia in a minority of ectopic cases with prolonged GHRH exposure can also progress to a true pituitary adenoma [81], [91], [92]. The finding of pituitary hyperplasia should precipitate investigations to localize an extra-pituitary NET [91]. This effect of prolonged GHRH stimulation causing pituitary hyperplasia is observed to be reversible following resection of the primary tumor or treatment with somatostatin receptor ligands (SRLs) [22], [70], [93], [94].

Sano et al. reported hyperprolactinemia in 16 out of 30 cases of GHRH-ectopic acromegaly with no relation to MEN1 while in the review by Ramirez et al. 18.1% of the tumors were found to be related to MEN1 [81], [95].

Pancreatic NETs causing ectopic GHRH-related acromegaly are more inclined to secrete other hormones such as insulin, gastrin, glucagon, pancreatic polypeptides, vasoactive intestinal peptide, adrenocorticotropic hormone (ACTH), calcitonin, chromogranin, serotonin or catecholamines [68], [70], [81], [85], [96], [97], [98]. Ramirez et al. reported 40% of pancreatic and 8% of the lung NETs with ectopic GHRH production and hyperprolactinemia co-secreted other hormones [81].

Significant pituitary hyperplasia in ectopic GHRH secretion can also lead to pituitary apoplexy as demonstrated in a case of GHRH-producing bronchial NET associated with pituitary somatotroph and lactotroph hyperplasia, where rapid shrinkage of the pituitary gland was seen after apoplexy and resection of the primary bronchial NET [79].

### Additional dynamic testing

Given that some ectopic tumors have a paradoxical response to Thyrotropin-releasing hormone (TRH), oral glucose administration, insulin-induced hypoglycemia i.e., an increase in GH of >50% from baseline, and blunted response to exogenous GHRH administration i.e., no rise in GH levels, these tests have been considered as additional dynamic tests for ectopic acromegaly [21], [95], [96], [99], [100], [101].

Sometimes, GH-secreting pituitary macroadenomas also show this paradoxical response to TRH and glucose administration hence limits the use of these provocative tests [102]. Additionally, the presence of some medications and diseases also makes the interpretation difficult. TRH stimulation in some cases may lead to pituitary apoplexy, is contraindicated in pregnancy, and has potential risks associated with rapid increase in thyroid hormone levels and is not approved by the United States Food and Drug Administration [81], [103], [104], [105].

### Use of Imaging – from CT scans to molecular & hybrid imaging

Once a pituitary tumor is ruled out after biochemical diagnosis of acromegaly, the ectopic source should be localized by imaging techniques. Most tumors arise from the thorax and abdomen therefore can be identified by conventional imaging such as contrast-enhanced computed tomography (CT) scan of the chest and abdomen [85]. If no tumors are found, then MRI can be used as 2nd line imaging modality to detect smaller masses.

Tumors causing acromegaly are usually well-differentiated and express somatostatin receptors (SSTRs) particularly SSTR subtype 2, therefore somatostatin receptor scintigraphy (SRS) or Octreoscan using radiolabeled somatostatin analogue 111Indium-pentetreotide diethylenetriaminopentaacetic (111In -DPTA-pentetreotide) can be used for localization of these tumors [85], [106]. Overall 111In -DPTA-pentetreotide scintigraphy has a sensitivity of 80–100% in well differentiated neoplasms but is lower in poorly differentiated NETs [107].

Positron emission tomography (PET) scan coupled with octreotide-labelled radioisotopes such as 68Gallium DOTATATE (68Ga-DOTATATE) is considered superior in detecting smaller NETs as compared to octreoscan as demonstrated in a patient with pancreatic NET which was not detected by 111In-DPTA-pentetreotide scintigraphy [67], [108]. In a study by Schreiter et al. 68Ga-DOTATATE PET/CT was able to detect more primary NETs as compared to 111In-DPTA-pentetreotide scintigraphy (45.5% vs 8%, *p* < 0.001) [109]. The use of 68Ga-DOTATATE PET/CT is considered a standard but is constrained by practical and economic challenges. An emerging alternative somatostatin analogue is 18Flourine-labelled octreotide, and non-inferiority of 18Flourine 1,4,7- triazacyclononane-N,N′,N″-triacetic acid octreotide (18F-AlF-NOTA-OC) to 68Ga- DOTATATE was demonstrated in a prospective multicenter trial [110], [111]. 18Fluoro-deoxyglucose PET (18FDG-PET) can also be used in poorly differentiated tumors if all other imaging techniques are unyielding. Its use is limited due to its poor uptake by well-differentiated NETs [112].

99Technetium-Hydrazinonicotinamide-Tyr3-octreotide (99Tc-HYNIC-TOC) is another modality that can be combined with single-photon emission computed tomography (SPECT) which gives higher image quality and lower radiation dose than Octreoscan [107]. Koçyiğit Deveci et al. demonstrated that 99Tc-HYNIC- TOC SPECT/CT was able to detect 40/43 lesions as compared to octreoscan which detected 36/43 lesions, however, the difference was not found to be statistically significant [113].

Despite availability of these imaging modalities sometimes the localization of the ectopic source remains unidentified. In a French series of 21 cases of ectopic GHRH-related acromegaly, the tumor was not revealed by CT scans of the thorax and abdomen, SRS, and 18FDG-PET scan [85]. Sometimes endoscopic sonography can also provide additional information such as tumor size and depth of invasion and can also be used for obtaining samples for biopsy [81], [85]. 11Carbon-methionine-PET (11C-Met PET) is another emerging modality which can be used in cases of occult NETs causing acromegaly, however, limited availability of the tracer limits its use [83], [114].

### Management of ectopic hormone secretion

There are no specific guidelines for treatment of ectopic acromegaly. Once ectopic acromegaly is diagnosed biochemically, and the source is localized by imaging techniques, surgical resection of the tumor and when feasible of the metastases should be considered the treatment of choice. However, comorbidities and overall health of the patient, extent and burden of the disease, and available resources play a pivotal role in determining the candidacy of the patient for surgical treatment [66], [81], [106].

Those patients who are poor surgical candidates due to disseminated metastatic disease or poor health status can benefit from somatostatin receptor ligand (SRL) therapy which can also be used as adjuvant therapy for patients who undergo surgery. SRLs can normalize or reduce IGF-1 levels but do not have significant effects on the size of tumor lesions in ectopic acromegaly [22], [66].

Post-operative GHRH levels are considered good indicators of tumor status in patients with GHRH-related ectopic acromegaly. Undetectable levels after surgical resection indicate remission while persistent levels are seen in residual disease [22]. Additionally, GHRH levels are also used to monitor recurrence in the postoperative period [85]. In contrast, GHRH levels decline but do not normalize with SRL therapy, but they can still be utilized to monitor the treatment response as a rise in GHRH levels can occur before recurrence of characteristic clinical symptoms [85], [115], [116]. Monitoring IGF-1 levels and oral glucose tolerance testing for GH suppression can also be helpful.

In case of a partial treatment response to SRLs, adding pegvisomant can help achieve normalization of biochemical markers and amelioration of symptoms of acromegaly [117].   Another emerging therapeutic option is peptide receptor radionuclide therapy with 177Lutathera-DOTATATE (177Lu-DOTATATE) for somatostatin receptor-positive NETs including GH- or GHRH-secreting NETs particularly when unresectable or metastatic. 177Lu-DOTATATE binds to SSTR receptors particularly SSTR type 2, and delivers targeted β-radiation, potentially reducing tumor burden and ectopic hormone secretion [25].

Other treatment options include conventional anti-neoplastic therapies such as chemotherapy, radiotherapy, immunotherapy, chemoembolization of the metastases, other hormonal therapies such as bromocriptine and cabergoline and targeted therapies [22], [66], [118], [119]. The use of these options varies according to the type of the organ involved, staging of the tumor and response to previous therapies, and is beyond the scope of this review.

## CRediT authorship contribution statement

**Maham Shahid:** Writing – original draft, Investigation, Formal analysis, Data curation. **Christian A. Koch:** Writing – review & editing, Supervision, Conceptualization.

## Declaration of competing interest

The authors declare the following financial interests/personal relationships which may be considered as potential competing interests:

Christian A Koch reports a relationship with Elsevier that includes: consulting or advisory. Associate Editorship: JCTE, Journal of the Endocrine Society. Sub Principial Investigator: Study by Abbvie Inc. If there are other authors, they declare that they have no known competing financial interests or personal relationships that could have appeared to influence the work reported in this paper.
